# Non‐lethal loop‐mediated isothermal amplification assay as a point‐of‐care diagnostics tool for *Neoparamoeba perurans*, the causative agent of amoebic gill disease

**DOI:** 10.1111/jfd.13175

**Published:** 2020-05-04

**Authors:** Irene Cano, Robin McCullough, Brian Mulhearn, Susie Gunning, Ava Waine, Claire Joiner, Richard Paley

**Affiliations:** ^1^ International Centre of Excellence for Aquatic Animal Health Cefas Weymouth Laboratory Weymouth UK

**Keywords:** amoebic gill disease, loop‐mediated isothermal amplification, *Neoparamoeba perurans*, point‐of‐care test

## Abstract

*Neoparamoeba perurans* is the causative agent of amoebic gill disease (AGD). Two loop‐mediated isothermal amplification (LAMP) assays targeting the parasite 18S rRNA and the Atlantic salmon EF1α, used as internal control, were designed. The *N. perurans* LAMP assay did not amplify close relatives *N. pemaquidensis* and *N. branchiphila*, or the host DNA. This assay detected 10^6^ copies of the parasite 18S rRNA gene under 13 min and 10^3^ copies under 35 min. Five “fast‐and‐dirty” DNA extraction methods were compared with a reference method and further validated by TaqMan™ qPCR. Of those, the QuickExtract buffer was selected for field tests. Seventy‐one non‐lethal gill swabs were analysed from AGD‐clinically infected Atlantic salmon. The pathogen was detected under 23 min in fish of gill score >2 and under 39 min for lower gill scores. About 1.6% of the tests were invalid (no amplification of the internal control). 100% of positives were obtained from swabs taken from fish showing gill score ˃3, but only ~50% of positives for lower gill scores. The present LAMP assay could be implemented as a point‐of‐care test for the on‐site identification of *N. perurans*; however, further work is required to improve its performance for lower scores*.*

## 1 INTRODUCTION

Amoebic gill disease (AGD) is a worldwide distributed disease affecting the salmonid industry in the marine environment (Nowak & Archibald, 2018). AGD has also been reported in other non‐salmonid species, such as the cleaner fish ballan wrasse *Labrus bergylta* (Ascanius, 1767) among others (Oldham, Rodger, & Nowak, 2016).

The causative agent of AGD is the free‐living protozoan *Neoparamoeba perurans* [see Ref. (Young, Crosbie, Adams, Nowak, & Morrison, 2007)], which can colonize the gills, and the resulting host response causes disease. Characteristic AGD clinical signs in Atlantic salmon, *Salmo salar* (Linnaeus, 1758), are multifocal white mucoid spots and patches on the gill surface. Histologically, *N. perurans* causes hyperplasia and hypertrophy of the epithelial and mucous cells, leading to lamellar fusion, generally in association with attached amoebae (Adams, Ellard, & Nowak, 2004). Severe infections induce reduced feed intake, increased stress and gasping. Mortalities can reach up to 50% in the absence of treatments (Munday, Foster, Roubal, & Lester, 1990).

AGD can be proactively managed by regular inspection of the gills of anaesthetized fish for gross AGD signs. A “gill index” is used internationally, with a scale from 0 = no lesions to 5 = extensive lesions, by examining all the hemibranch surfaces (Noguera et al., 2019). The gill index allows the farmer to plan treatments in a cost‐effective manner (Taylor, Muller, Cook, Kube, & Elliott, 2009). However, other pathogens can cause gill disorders in marine‐farmed salmon, and can present as bleeding gills, pale/thickened patches on the gills, focal lesions and necrosis of the gill lamellae and rakers. Those gill disorders may be multifactorial and might include phytoplankton, parasites, jellyfish, algal blooms, bacteria and viruses (Baxter, Rodger, McAllen, & Doyle, 2011;Rodger, Henry, & Mitchell, 2011;Rodger & Mitchell, 2007). The gill score is therefore presumptive, a confirmatory test of the presence of *N. Perurans* is desirable before treatments are conducted. For the confirmation of *N. perurans*, a wet mount of gill smear can be done at the farm site for trained staff for the presence of amoebic cells; however, other morphologically similar *Neoparamoeba s*pecies, such as *N. pemaquidensis* and *N. branchiphila*, which are not pathogenic, can be isolated from gill smears and confounded as the aetiological agent of AGD (Young et al., 2014). Histology assessments can also confirm the presence of amoebas in association with gill lesions, but it is time‐consuming and difficult to carry out on‐site.

The targeted tissue for the AGD infection allows for non‐destructive sampling based on gill swabs. A strong correlation of the pathogen identification by molecular methods, gill score and histopathology scores has been demonstrated (Downes et al., 2017). Available PCR‐based methods, all of them targeting the parasite 18S rRNA gene, can be used to confirm the presence of *N. perurans* (Bridle, Crosbie, Cadoret, & Nowak, 2010; Downes et al., 2017; Fringuelli, Gordon, Rodger, Welsh, & Graham, 2012; Young, Dyková, Nowak, & Morrison, 2008); however, those tests are time‐consuming and require a laboratory and trained staff.

Loop‐mediated isothermal amplification (LAMP) amplifies nucleic acids with high specificity, sensitivity and rapidity under isothermal conditions. The assay uses DNA polymerase with high strand displacement activity and a set of six specific primers on the target DNA to achieve highly selective nucleic acid amplification (Notomi et al., 2000). DNA can be amplified 10^9^‐ to 10^10^‐fold in 15–60 min. There is no requirement for the temperature cycling of conventional polymerase chain reaction amplification; therefore, assay times are reduced. Additionally, due to its ability to amplify nucleic acid under isothermal conditions simple and low‐cost equipment can be used (Sahoo, Sethy, Mohapatra, & Panda, 2016). Several LAMP assays have been evaluated for the identification of aquaculture pathogens, including bacterial, viral and parasitic pathogens causing serious diseases in aquaculture (Biswas & Sakai, 2014). These tests present the potential of using LAMP for point‐of‐care (POC) tests; however, effective POC DNA extraction methods, based on “fast‐and‐dirty” DNA extractions, are also essential to develop rapid and user‐friendly molecular diagnostic assays for field sampling (Lau & Botella, 2017).

In this study, a LAMP assay, using real‐time fluorescence equipment, was evaluated for the detection of *N. perurans* on swabs taken non‐lethally from Atlantic salmon gills. Furthermore, several POC DNA extraction methods were compared for field applications. Finally, the chosen protocol underwent preliminary field testing, and the results were compared with visual gill scores.

## 2 MATERIALS AND METHODS

### 2.1 *Neoparamoeba perurans* and *N. pemaquidensis* culture

*Neoparamoeba perurans* trophozoites were isolated from the gills of naturally infected Scottish farmed sea‐cage Atlantic salmon showing typical AGD lesions as described previously (Morrison, Crosbie, & Nowak, 2004). The related *N. pemaquidensis*, sourced from the culture collection ATCC‐50172 (LGC Standards), and the isolated *N. perurans* were cultured on malt yeast agar (MYA: 0.01% malt, 0.01% yeast, 2% Bacto agar, 0.2 µm of filtered sea water (SW) at 35‰ salinity) overlaid with 0.2 µm of filtered SW. Plates were incubated at 18°C and amoebae passaged fortnightly by transfer of SW to fresh MYA plates with an additional overlay of 0.2 µm of filtered SW (Crosbie, Bridle, Cadoret, & Nowak, 2012).

### 2.2 DNA extractions from amoebic culture

Genomic DNA was extracted either from Isohelix DNA Buccal Swabs (Sigma‐Aldrich, UK) soaked in the in vitro culture of *N. perurans*, or from aliquots of *N. pemaquidensis*, and aliquots of serial dilutions of *N. perurans* cells were counted in a TC20 automated cell counter (Bio‐Rad, UK). Cells were pelleted by centrifugation at 18,000 ×g for 10 min, resuspended in the digestion buffer G2 and incubated at 56°C with proteinase K (600 mAU/ml) for at least 1 hr. Soaked swabs were placed in 200 µl of the G2 buffer and incubated with proteinase K as described before. DNA was then extracted using the EZ1 DNA Tissue Kit and an EZ1 extraction robot (Qiagen) following the manufacturer's protocol.

DNA from *N. branchiphila* was sourced from the University of Tasmania (Australia).

In addition, DNA from Atlantic salmon gill homogenates (1:10 weight/volume in G2 buffer) was extracted and used as a negative control.

### 2.3 LAMP assay design

A specific LAMP assay was designed targeting the *N. perurans* 18S rRNA gene. A multiple sequence alignment against the 18S rRNA genes of available *N. perurans* sequences (GenBank accession numbers: EF216900.1, EF216902.1, EF216904, EF216905, GQ407108, GU574794, KF146712.1, KF179520.1, KT989880.1, KT989881.1, KU985057.1 and KU985058.1) was carried out using MegAlign v7.0.21 (Lasergene, DNASTAR) to identify conserved sequences. Primers were then designed in those areas using the LAMP Designer 1.10 program (Premier Biosoft International) consisting of two outer primers (F3 and B3), two inner primers (FIP and BIP) and two loop primers (Loop‐F and Loop‐B) (Table 1), targeting a region of 343 bp. The specificity of the primers was tested in silico against the Atlantic salmon 18S rRNA gene (AJ427629.1) using a ClustalW alignment analysis.

**Table 1:** Sequences of primers designed for the *Neoparamoeba perurans* 18S rRNA gene and the Atlantic salmon elongation factor 1 alpha LAMP assays

LAMP assay	Primer	Sequences (5’−3’)
*N. perurans* 18S	F3	TGAGTGATAAGCAGACCTATTG
	B3	TTCGCAGAAGTTCGTCTT
	FIP	TTGCTTGCCTTGAACACTCTAAGGTTTAAGATTGTGGAGGTTCT
	BIP	TTTTCGGAGAGAGATGAAGTGTATCCAAGAATTTCACCTCTG
	Loop‐F	ACTGAATCTAAGCAGAACGAAC
	Loop‐B	GGGCATTCGTATTTCATTGT
Atlantic salmon EF1α	F3	AGACTGGCAGGTACTACG
	B3	CTTGATGTAGGTGCTGACC
	FIP	ACTCACCAACACCACCAGC|AGAACATGATCACT
	BIP	CGTGAGCACGCACTCCTT|TCTGTGGAGTCCATCT
	Loop‐F	CGATAAGCACAGCACAATCAG
	Loop‐R	GAGTGAAGCAGCTCATCGT

A second LAMP assay to amplify a region of 295 bp on the Atlantic salmon elongation factor 1 alpha (EF1α) (NM_001123629.1) was designed as described above and used as an internal control (Table 1).

### 2.4 Recombinant plasmid

A fragment of 1632 bp of the *N. perurans* 18S rRNA gene containing the LAMP probing region was amplified using the set of primers Generic 1F (5’‐TATGGTGAATCATGATAACTTWAC‐3’) and B3‐Z (5’‐GGAATTCCTCGTTCACGATAA‐3’) and cloned into the pGem‐T Easy plasmid vector (Promega). The template (dsDNA) copy number was calculated using a QuantiFluor dsDNA kit in a Quantus fluorimeter (Promega), and a plasmid dilution series, from 10^6^ to 1 copy, was generated to obtain a standard curve.

### 2.5 Assay optimization

The reaction temperature was optimized using a block gradient from 60 to 68°C at 0.1°C intervals followed by an annealing step of 98–80°C, ramping at 0.05°C per second. LAMP reactions contained 15 μL of the fast isothermal master mix (ISO‐004, OptiGene), 5 pmol of each primer F3 and B3, 10 pmol of each Loop‐F and Loop‐R, 20 pmol of FIP and BIP, either 10^5^ copies of the recombinant plasmid or 5 μl of the extracted DNA and nuclease‐free water to a final volume of 25 μl.

When DNA was extracted using KOH (as described below), the isothermal Lyse'n’LAMP master mix (ISO‐001LNl, OptiGene) was used instead.

Isothermal amplification was performed either in a Genie® II or a Genie® III system (OptiGene) for real‐time monitoring of the LAMP amplification. The amplification ratio measured as the change of fluorescence over time and expressed as the time of positivity (*Tp*), and the amplicon annealing temperature were analysed using a Genie® II or a Genie® III software (OptiGene).

### 2.6 Specificity and sensitivity of the LAMP assay

The specificity of the *N. perurans* LAMP assay was assessed against close relatives *N. pemaquidensis* and *N. branchiphila*, as well as host (Atlantic salmon) DNA.

A total of 10‐fold serial dilutions of the recombinant plasmid, ranging from 10^6^ to 1 copy, were used to determine the limit of detection (LOD) of the assay. Linear regression analysis between the number of copies and *Tp* was performed from three different independent assays.

### 2.7 “Fast‐and‐dirty” DNA extraction protocols

Isohelix swabs were soaked in the *N. perurans *in vitro culture and DNA‐extracted following either “fast‐and‐dirty” DNA extraction methods as described below or the reference laboratory method using the EZ1 DNA tissue Kit.

Five “fast‐and‐dirty” DNA extraction methods for POC testing were evaluated: sodium hydroxide (NaOH), QuickExtract™ DNA extraction solution (Cambio, UK), potassium hydroxide (KOH), KAPA Express Extract (Sigma, UK) and Buccalyse DNA release (BEK‐50, Isohelix, UK), and compared with the reference laboratory method. Five swabs per protocol were tested in duplicate by LAMP assay and TaqMan™ qPCR.

The NaOH protocol was adapted from previously published works (Truett et al., 2000;Valverde, Cano, Castro, Paley, & Borrego, 2017). *N. perurans*‐soaked swabs were placed in 475 μl of 50 mM NaOH (pH 12) lysis reagent and incubated for 10 min at 95ºC, and then, 25 μl of 100 mM of Tris‐hydrochloric acid (pH 5) was added to neutralize the lysis reaction.

QuickExtract™ DNA extraction solution was used following the manufacturer's recommendations. Swabs were placed in 500 μl of the QuickExtract™ buffer and incubated for 6 min at 65°C followed by 2‐min incubation at 98°C.

For the alkaline KOH protocol, swabs were placed in a tube containing 250 μl of filtered SW and 250 μl of 600 mM KOH (pH13) and incubated for 5 min at 95°C followed by a brief cooling period on ice (OptiGene, 2018).

For testing the KAPA extraction kit, swabs were placed in 472 μl of water, 25 μl of KAPA buffer and 3 μl of KAPA enzyme, and incubated for 10 min at 75°C, followed by 5‐min incubation at 95°C as recommended by the manufacturers.

Finally, for the Buccalyse DNA kit, swabs were placed in 500 μL of Buccalyse and incubated for 15 min at 70°C followed by an incubation of 2 min at 95°C following the manufacturer's instructions.

The amount of the extracted DNA obtained with the different POC methods was measured using a NanoDrop ND‐1000 spectrophotometer (Labtech).

### 2.8 Gill swabs from naturally infected Atlantic salmon

To validate the selected POC DNA extraction method, 11 naturally AGD‐infected Atlantic salmon, of approximately 200g, were collected from an open‐water pen from Northern Scotland. Gill swabs were taken in duplicate from the anaesthetized fish by swabbing the gills. For each animal, one swab was DNA‐extracted with the laboratory reference protocol using the EZ1 robot extraction and the second swab was DNA‐extracted using the QuickExtract protocol. A visual gill index, ranging from 0 to 5, was recorded for each animal (unaffected gills: gill score 0; to severe lesions covering the majority of the gill area: gill score of 5) as the average of the 16 hemibranch surface (both sides of all 8‐gill arches) scores (Taylor et al., 2009). The identification of *N. perurans* from the swabs was analysed both by TaqMan™ qPCR (as described below) and by LAMP assay.

### 2.9 Gill swabs from challenged Atlantic salmon

To generate AGD‐positive gill swabs of Atlantic salmon with low visual gill score, an AGD bath challenge was carried out as described previously (Cano et al., 2019). Briefly, two tanks containing 45 Atlantic salmon reared in the biosecure stock aquarium areas of the Cefas Weymouth Lab from ova, weighing approximately 200g, were exposed to *N. perurans* by static bath immersion using either 2,500 or 500 trophozoites per L^1^ for 4 hr, respectively. Then, the flow rate was restored to 5–7 L per minute and the water temperature was maintained at 12 ± 1°C. Fish were examined from the high dose at day 22 to the low dose at day 29 after challenge, and then weekly to follow the disease progression. The visual gill index was noted, and a single gill swab per animal was taken. DNA was extracted from a total of 60‐gill swabs using the QuickExtract protocol, and the presence of *N. perurans* was analysed by LAMP assay. A positive control using either 10^5^ copies of the recombinant plasmid or DNA extracted from positive AGD‐infected fish was run alongside the tests. A LAMP assay to amplify the Atlantic salmon EF1α was carried out in parallel. Any test with no amplification of the host DNA was considered an invalid test.

An additional 12 swabs, taken from the specific‐pathogen‐free Atlantic salmon, were used as non‐infected control samples.

### 2.10 Result validation by TaqMan™ qPCR

A real‐time TaqMan™ qPCR (Fringuelli et al., 2012) was compared with the LAMP results when testing the POC extraction methods. TaqMan™ assays were performed with 5 μL of the extracted DNA, 500 nM of each primer and 250 nM of probe labelled with 6‐FAM in 5′ and MGB in 3’, in a total volume of 20 μL by using the TaqMan™ Universal PCR Master Mix with AmpErase UNG (Applied Biosystems). TaqMan™ qPCR assays were performed on a StepOnePlus™ Real‐Time PCR System (Life Technologies) at 50°C for 2 min followed by 95°C for 10 min, then 40 cycles of 15 s at 95°C and 1 min at 60°C. Each sample was tested in duplicate.

In addition, the qPCR assay was used to estimate the number of copies of the 18S rRNA gene in a single amoebic cell. DNA was extracted from 10‐fold serial dilutions of amoebic cells, containing 10^3^ to 1 cell, and the CT values were correlated with the number of copies of the 18S rRNA gene in a standard curve.

### 2.11 Ethics statement

Animal procedures were approved by the Animal Welfare and Ethical Review Body (AWERB) at the Cefas Weymouth Laboratory and conducted in compliance with the Animals (Scientific Procedures) Act 1986.

## 3 RESULTS

### 3.1 LAMP assay optimization

**Figure 1:**
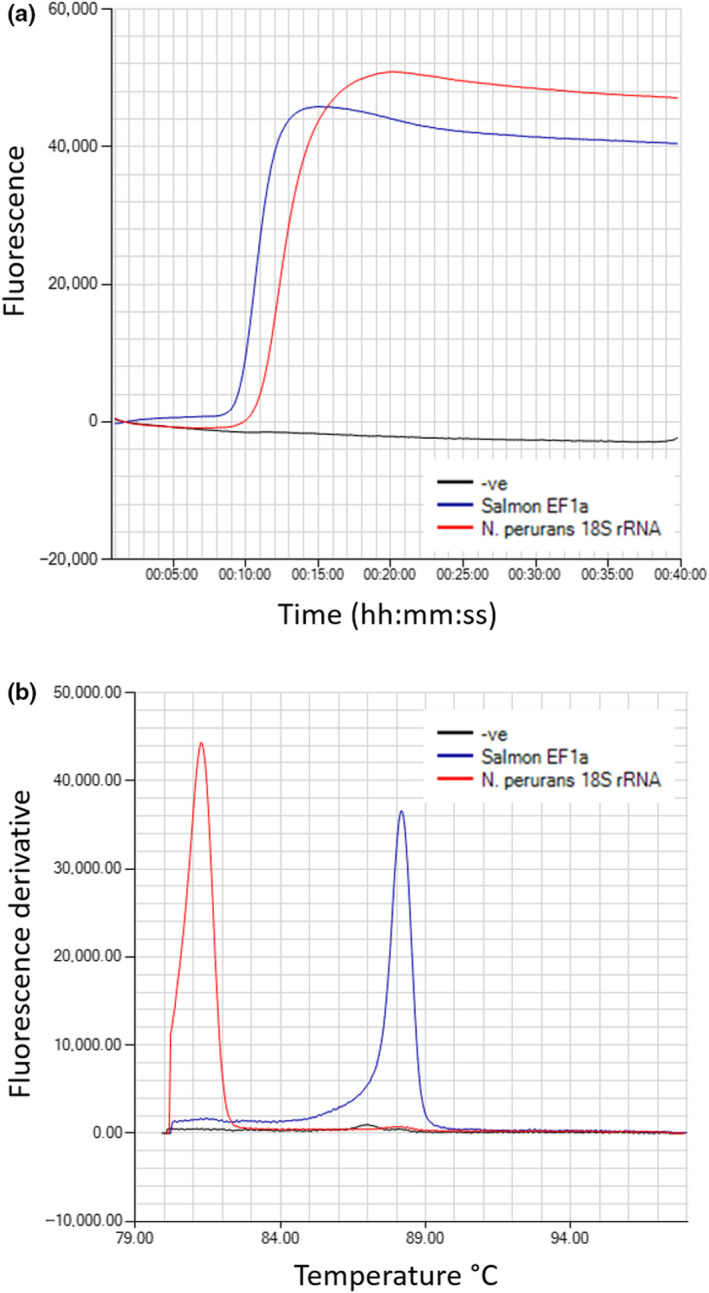
*Neoparamoeba perurans* 18S rRNA gene and Atlantic salmon EF1α LAMP assays. (a) Isothermal amplification. (b) Anneal derivative of isothermal amplified products [Colour figure can be viewed at wileyonlinelibrary.com]

Testing temperatures from 60 to 68°C resulted in the selection of the optimal amplification temperature (faster detection) of 62.9°C for the *N. perurans* LAMP assay. For standardization purposes, the internal control Atlantic salmon EF1α LAMP assay was run at the same temperature as the *N. perurans* LAMP assay. The annealing curves of the amplified products for the *N. perurans* LAMP assay, using either the recombinant plasmid or extracted DNA, showed a single peak in the range of 81–82°C, while the annealing temperature for the Atlantic salmon LAMP assay was 88°C (Figure 1).

### 3.2 *N. perurans* LAMP assay specificity and analytical sensitivity

**Figure 2:**
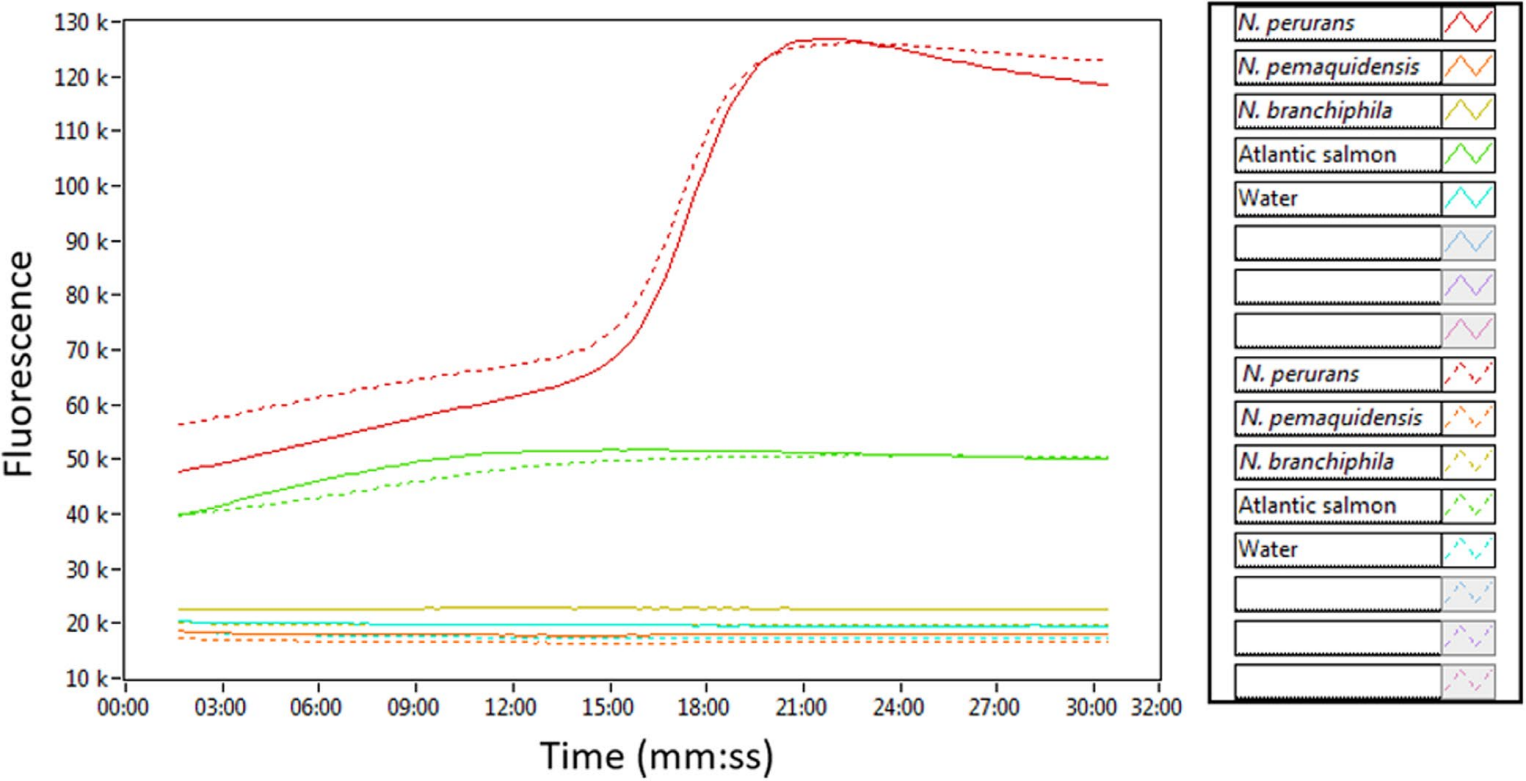
The specificity of the *Neoparamoeba perurans* LAMP assay. Isothermal amplification of DNA extracted from *Neoparamoeba perurans*, *N. pemaquidensins*, *N. branchiphila* and Atlantic salmon, each species in duplicate. Water was used as a negative control [Colour figure can be viewed at wileyonlinelibrary.com]

The *N. perurans* LAMP assay did not amplify close relatives *N. pemaquidensis* and *N. branchiphila*, or Atlantic salmon DNA from tissue homogenates (Figure 2).

**Figure 3:**
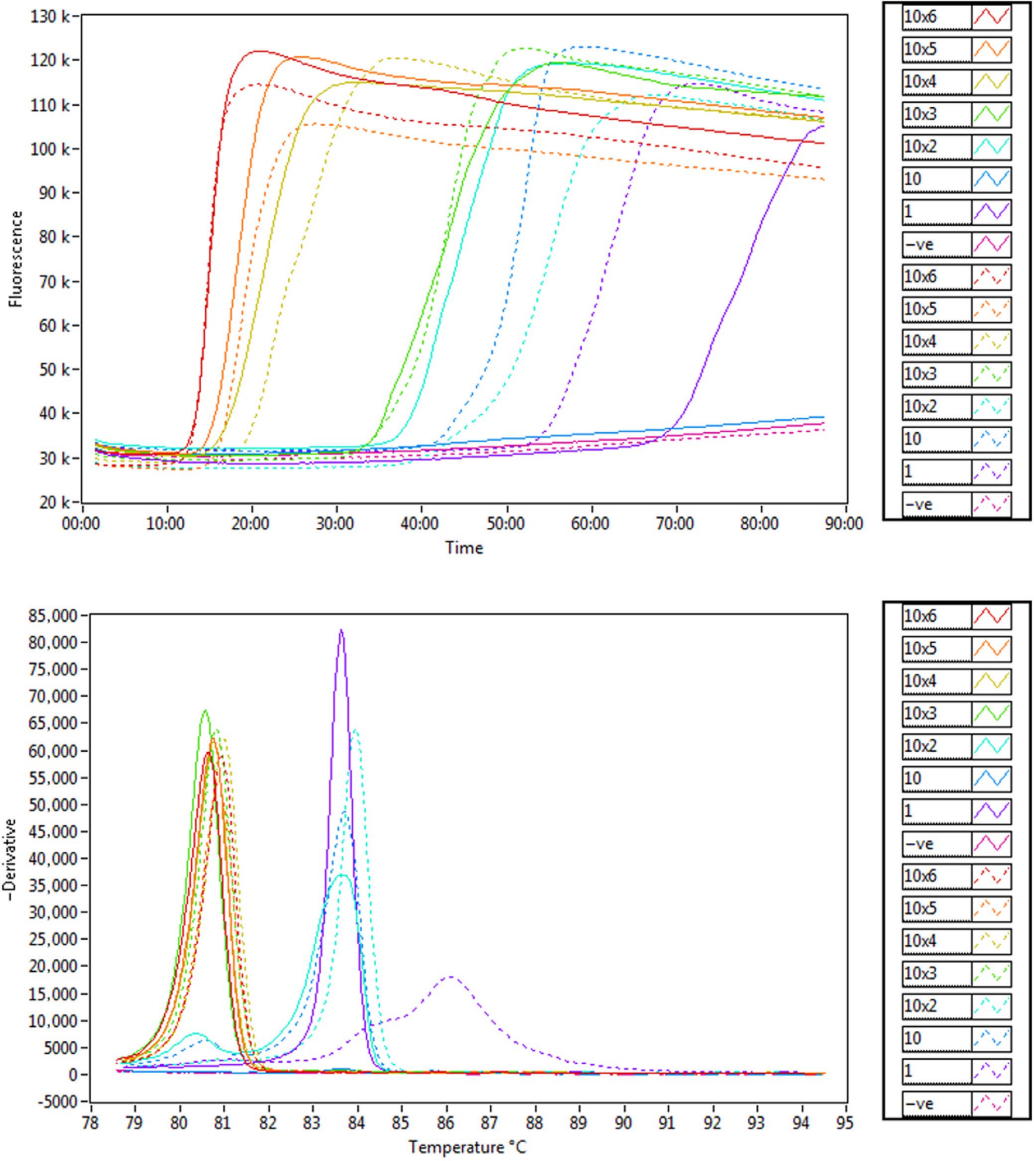
The **s**ensitivity of the *Neoparamoeba perurans* LAMP assay. (a) Amplification graph of serial dilutions ranging from 10^6^ to 1 copy of a recombinant plasmid. Each dilution was run in duplicate. (b) Anneal derivative of isothermal amplified products [Colour figure can be viewed at wileyonlinelibrary.com]

**Figure 4:**
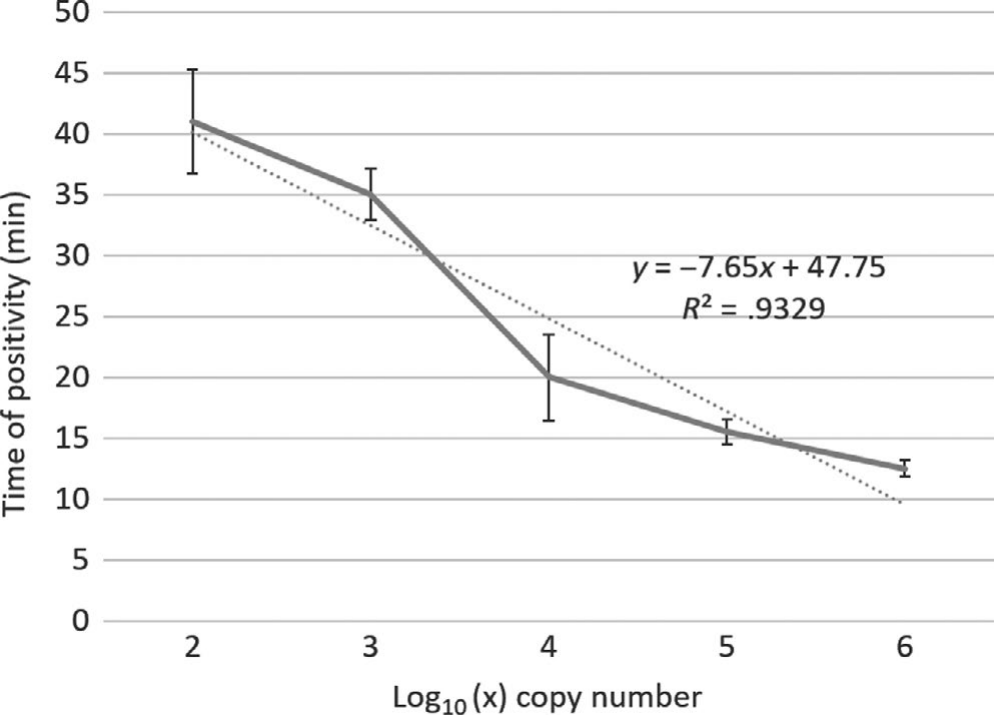
Linear correlation of the *Neoparamoeba* perurans LAMP assay between the plasmid copy number (expressed as Log10(*x*)) and the time of positivity (*Tp*). Mean data of three independent assays

Taking the average of 3 independent runs, the assay detected 10^6^ copies of the recombinant plasmid under 13 min and 10^3^ copies under 35 min. Dilutions containing 100 copies showed inconsistent results, with the generation of a second amplicon at a different annealing temperature than the expected at 81–82°C, probably due to the amplification of secondary structures as a result of low amounts of the target sequence or primer dimers. Either both 10 and 1 copies of the template failed to amplify the target or the amplicon showed a different annealing temperature (Figure 3). Therefore, 10^3^ copies were accepted as LOD for the assay, and a test run of 40 min was established for later analysis. Linear regression analysis showed a strong correlation (Pearson's* r* = −.96) between the number of the recombinant plasmid copies and *Tp* for 10‐fold dilutions between 10^6^ and 100 copies (*r* ^2^ = .93) (Figure 4).

Linear regression analysis carried out on a TaqMan™ qPCR assay between the number of amoebic cells and a plasmid copy standard curve (Figure S1) gave an estimated average number of 856 copies of the 18S rRNA gene per cell.

### 3.3 POC DNA extraction methods

All the POC DNA extraction methods yielded detectable amounts of DNA when extracted from amoeba‐soaked swabs, except the NaOH method, which only extracted DNA from 1 out of the five swabs tested. The QuickExtract showed the purest DNA extraction (absorbance ratio 260/280 closest to 1.8) after the EZ1 Biorobot reference laboratory method. All the methods showed a low ratio of 260/230, indicating the presence of contaminants in the DNA extraction.

**Figure 5:**
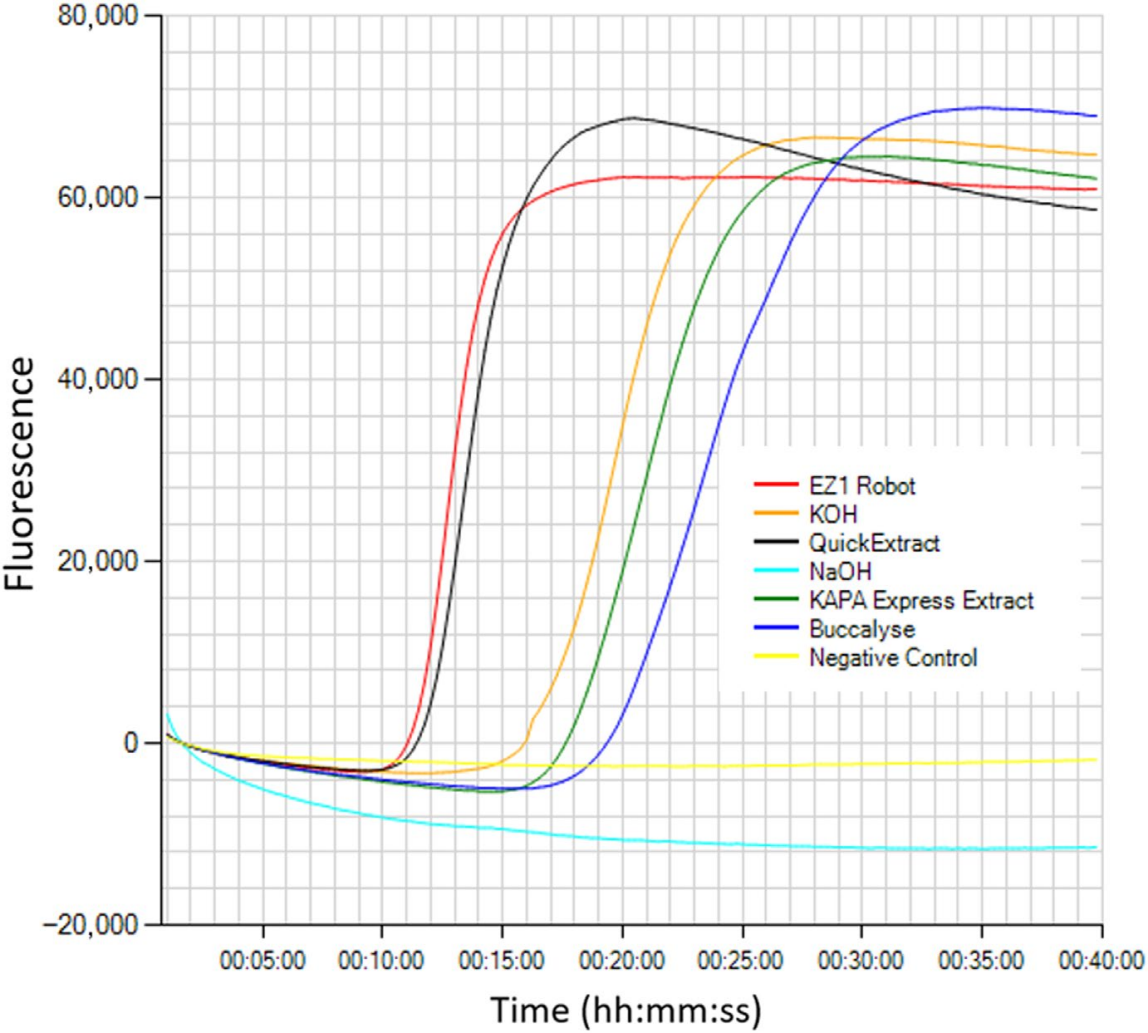
Isothermal amplification of *Neoparamoeba perurans* from Isohelix swabs. DNA was extracted either with: NaOH, QuickExtract, KOH, KAPA Express Extract or Buccalyse DNA release, and compared with a reference laboratory method (EZ1 Biorobot) [Colour figure can be viewed at wileyonlinelibrary.com]

DNA extracted with the QuickExtract method ranked the fastest LAMP amplification of all the five POC methods tested, with a *Tp* of 18:45 mm:ss (average of five Isohelix swabs) versus 12:45 for the laboratory reference method. DNA extracted with the QuickExtract buffer was also compatible with the parasite detection by TaqMan™ qPCR chemistry, allowing for laboratory validation of field LAMP results (Figure 5, Table 2).

**Table 2:** Comparison of five point‐of‐care DNA extraction methods: NaOH, QuickExtract, KOH, KAPA Express Extract and Buccalyse DNA release with a reference laboratory method (EZ1 Biorobot) for the LAMP detection of *Neoparamoeba perurans* using cultured amoeba‐soaked Isohelix swabs

DNA extraction method	DNA ng/ul	Ratio 260/280	LAMP		TaqMan™ qPCR	
			*Tp* (mm:ss)	+ve	*Ct*	+ve
EZ1 Biorobot	0.5 ± 1.2	1.8	12:45 ± 0:50	5/5	30.7 ± 3.1	5/5
NaOH	28.1	1.5	Undetectable	0/5	Undetectable	0/5
QuickExtract	38.8 ± 14.6	1.7	18:45 ± 4:35	5/5	36.5 ± 1.4	5/5
KOH	200.9 ± 38.3	1.5	33:15 ± 4:15	3/5	Undetectable	0/5
KAPA Express Extract	32.3 ± 3.6	1.3	22:45 ± 3:00	5/5	Undetectable	0/5
Buccalyse DNA release	52.7 ± 6.5	1.1	20:50 ± 2:10	3/5	36.5 ± 0.5	3/5

Note

Number of positive samples (+ve) expressed as number of positive swabs/total number of swabs analysed per method. LAMP amplification expressed as the time of positivity *Tp* (mm:ss). TaqMan™ qPCR detection expressed as cycle threshold (*Ct*) values.

The second fastest POC method was obtained with Buccalyse DNA release buffer, showing an average of *Tp* 20:50 for the detection of *N. perurans* in swabs, followed by KAPA Express Extract (*Tp* 22:45) and KOH method (*Tp* 33:15). DNA extracted with Buccalyse release was positive by TaqMan™ qPCR in only 3 out of 5 samples, while none of the samples extracted with KAPA Express Extract tested positive by qPCR. DNA extractions from swabs using NaOH failed to detect the parasite either by LAMP assay or by TaqMan qPCR. The QuickExtract POC method was then selected for field applications.

### 3.4 Comparison of *N. perurans* detection in gill swabs using the reference laboratory method and POC protocol

Two swabs per animal were analysed from 11 naturally infected AGD Atlantic salmon showing a visual gross gill score between 2 and 5. The *N. perurans* LAMP assay successfully detected the parasite in 100% of all the gill swabs when using the EZ1 Biorobot (reference method) independently of the gill score. Those LAMP‐positive tests were validated by TaqMan™ qPCR in all the samples. However, when using the QuickExtract (POC method), the parasite was detectable in 100% of the swabs taken from fish showing gill score of ≥3, but only in 33.3% (1 out of 3) of the swabs taken from fish of gill score of 2 (Table 3). From those two negative swabs extracted with the QuickExtract, of whom duplicate swab using the reference DNA extraction method tested positive by TaqMan™ qPCR, one of them also failed to detect the parasite by TaqMan qPCR, suggesting a poor DNA extraction. Therefore, to allow for the identification of false negatives due to the failure of the POC DNA extraction, the Atlantic salmon EF1α LAMP assay was run in parallel as an internal control in subsequent analysis.

**Table 3:** Comparison of *Neoparamoeba perurans* detection in gill swabs from naturally infected Atlantic salmon using a reference laboratory method (EZ1 Biorobot) and a point‐of‐care DNA extraction protocol (QuickExtract)

Gill score range	DNA extraction method	LAMP		TaqMan™ qPCR	
		*Tp* (mm:ss)	+ve	*Ct*	+ve
1–2	EZ1 Biorobot	18:15 ± 2:10	3/3	34.7 ± 0.9	3/3
	QuickExtract	20:15	1/3	39.2 ± 0.9	2/3
2.1–3	EZ1 Biorobot	16:15 ± 3:15	5/5	32.8 ± 3.4	5/5
	QuickExtract	19:25 ± 3:35	5/5	36.6 ± 3.2	5/5
3.1–4	EZ1 Biorobot	14:30 ± 2:25	2/2	33.5 ± 4.2	2/2
	QuickExtract	17:25 ± 1.0	2/2	37.5 ± 3.0	2/2
4.1–5	EZ1 Biorobot	12:38	1/1	28.8	1/1
	QuickExtract	19:15	1/1	38	1/1

Note

*N. perurans* detection was assayed by TaqMan™ qPCR (expressed as cycle threshold *Ct* values) and LAMP assay (expressed as time of positivity *Tp* (mm:ss)). Visual gill index expressed as the average of the 16 hemibranch scores. Number of positive samples (+ve) expressed as: number of positive swabs/total number of swabs analysed per gill score.

From those initial sampled fish, the *N. perurans* LAMP detection in swabs extracted with the reference laboratory method was significantly faster (*t* test, *p*‐value ˂ .024) than in swabs POC DNA‐extracted (*Tp* 16:30 ± 3:05 vs. 19:0 ± 2:5). The same was observed when analysed by TaqMan™ qPCR (*Ct* 33.1 ± 3.0 vs. 37.4 ± 2.6), *p*‐value ˂ .001.

Although a trend of a negative correlation between the visual gill score and the LAMP assay detection was observed (shorter *Tp* for higher gill score), the correlation coefficient was not significant for either of the DNA extraction methods (Pearson's* r* = −.57 for swabs DNA‐extracted with the EZ1 Biorobot and Pearson's* r* = −.22 with the QuickExtract method).

### 3.5 *N. perurans* POC detection in Atlantic salmon gill swabs showing low gill score

Sixty further swabs were taken from challenged Atlantic salmon showing a visual gill score between 0.13 and 3.58. DNA was extracted solely with the QuickExtract method, and both the *N. perurans* 18S rRNA gene and the Atlantic salmon EF1α, used as internal control, were analysed by LAMP assay (summarized in Table 4, raw data in Table S1).

**Table 4:** Point‐of‐care identification of *Neoparamoeba perurans* by LAMP assay

Gill score range	*N. perurans*		Atlantic salmon	
	*Tp*	*+ve*	*Tp*	+ve
0.1–1	32:50 ± 7:10	11/22	24:10 ± 6:0	21/22
1.1–2	35:30 ± 6:00	13/23	24:30 ± 5:10	23/23
2.1–3	29:00 ± 8:05	5/9	22:40 ± 4.45	9/9
3.1–3.6	29:05 ± 4:20	6/6	16:45 ± 1:40	6/6

Note

DNA was extracted from non‐lethal gill swabs using the QuickExtract method. The Atlantic salmon elongation factor 1a LAMP assay was used as an internal control. LAMP detection expressed as time of positivity *Tp* (mm:ss). The visual gill index expressed as the average of the 16 hemibranch scores. Number of positive samples (+ve) expressed as: number of positive swabs/total number of swabs analysed per gill score range.

An invalid test, failure to detect the Atlantic salmon EF1α gene, was observed in 1 sample out of the 60 swabs analysed (1.6%). *Tp* for the detection of the host EF1α ranged from 12 to 36 min.

**Figure 6:**
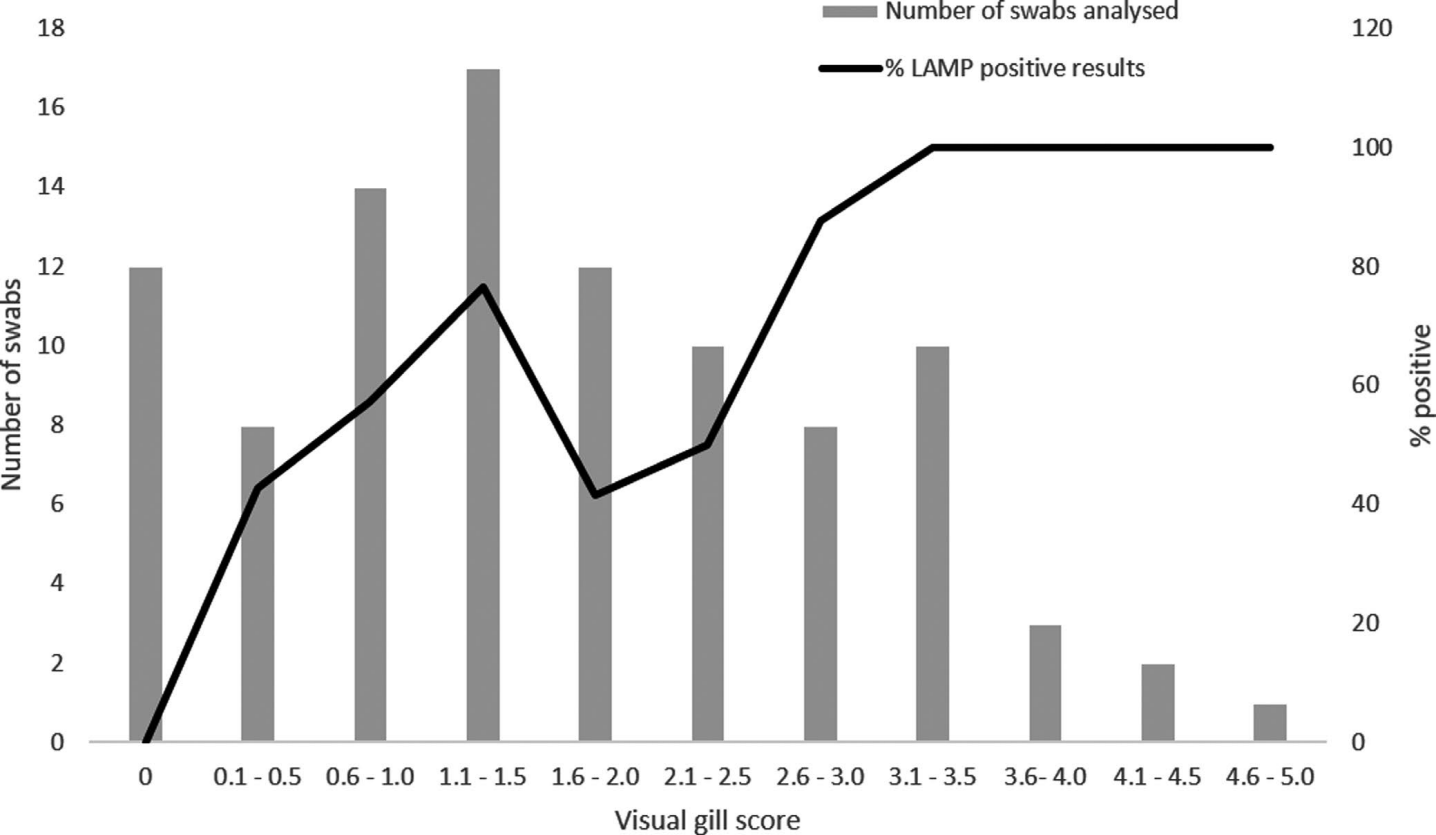
Summary of the number of Atlantic salmon gill swabs analysed (bars) and the percentage of LAMP‐positive tests (line) per gill score range. Bars include swabs taken both from naturally infected and from challenged fish

The POC LAMP assay confirmed the presence of *N. perurans* in 100% of the swabs taken from fish showing gill score ˃3. However, a decreased number of possitive samples from gill swabs of lower gill scores were observed (Figure 6), showing a strong correlation (*Pearson's r* = 0.86) between the positivity of the test and the gill score.

Times of detection ranged from 13 to 39 min. There was not a significant linear correlation between the times of detection and the visual gill scores (Pearson's* r* = −.23). *N. perurans* was not detected in any of the 12 negative control Atlantic salmon sampled.

## 4 DISCUSSION

An *N. perurans* LAMP assay has been evaluated for its application as a POC diagnostic test. Current industry practices rely on the visual gill score to plan cost‐effective treatments; however, a confirmatory test for the presence of the parasite is recommended before any treatment is given to the animals (Taylor et al., 2009).

The present *N. perurans* LAMP assay is a high specificity test that can discriminate, on‐site, the amoebic pathogen among other *Neoparamoebae* species that can colonize gills. The analytical sensitivity of the test (10^3^ copies of the parasite 18S rRNA gene) is similar to that of a conventional *N. perurans* diagnostic PCR, which can detect a LOD of 0.5 amoebae, equivalent to 2 pg of extracted DNA (Young et al., 2008). The *N. perurans* LAMP assay can thus be used to confirm the presence of the parasite in clinically infected fish. However, the analytical sensitivity of this assay is lower than expected for a real‐time fluorescence LAMP assay, which is typically comparable to a qPCR assay (McKenna et al., 2011). Published *N. perurans* qPCR assays reported a LOD within 3–100 copies of the 18S rRNA gene depending on the assay (Downes et al., 2017). In the present LAMP assay, the detection of a lower number of copies (˂100) of the *N. perurans* 16S rRNA gene required longer runs (more than 40 min) with the result sometimes yielding non‐specific amplicons (Ball et al., 2016). Currently, there is not a published genome of *N. perurans*; therefore, it is not known how many copies of the 18S rRNA gene a single amoeba might contain. In the present study, through serial dilutions of amoebic cells, an average number of 856 copies of the 18S rRNA gene per cell have been estimated. The amoeba *Hartmannella vermiformis* harbours an estimated average of 1,330 copies per cell (Kuiper et al., 2006), *Acanthamoeba* has approximately 600 copies, and *Naegleria* species seem to have several thousand copies (Qvarnstrom, Visvesvara, Sriram, & Da Silva, 2006). Thus, given the LOD of the LAMP assay, this test can potentially detect on‐site the presence of one amoeba in the extracted DNA.

To develop the LAMP assay as a POC test, five “fast‐and‐dirty” DNA extraction methods, three commercial kits and two house‐made buffers, were tested and compared with a reference laboratory DNA extraction method using positive *N. perurans* swabs. The robustness, compatibility with TaqMan™ qPCR chemistry for laboratory validation, simplicity and rapidity in the LAMP assay detection were compared across the five POC methods. The QuickExtract™ DNA extraction solution produced better results. This DNA extraction method has been used previously for the molecular characterization of amoebae of the genus *Flamella* and other aquatic microorganisms (Kotov & Taylor, 2010;Walthall, Tice, & Brown, 2016)*.* The complete POC DNA extraction protocol, from swabbing gills to LAMP ready‐extracted DNA, takes approximately 15 min, while the reference laboratory method typically takes a minimum of 1.25 hr (including the digestion step). However, when using the QuickExtract™ protocol, as well as the other POC DNA extraction methods tested, a reduced efficiency in the *N. perurans* LAMP assay, measured as a longer *Tp*, was observed when compared to the reference DNA extraction method. This is probably due to incomplete cell lysis (Heiniger et al., 2016). Those POC methods are based on chemical digestion to bring about the release of the nucleic acid. Currently, there are many other POC DNA extraction methods that have been tested for on‐site applications. Lateral flow devices (LFD) (Tomlinson et al., 2010), magnetic solid‐phase reversible immobilization (SPRI) (Lau & Botella, 2017) and microfluidics cartridges (Ali, Rampazzo, Costa, & Krieger, 2017), among others, have been reported as promising POC DNA extraction methods and could in future be implemented in commercial kits for the detection of *N. perurans* in the LAMP assay.

In developing a POC diagnostic kit, it is important to consider the presence of inhibitors during the DNA extraction method, which can then affect the test outcome (Ali et al., 2017). To detect invalid tests, an internal control based on the LAMP amplification of the host EF1α gene was run alongside the *N. perurans* test. Internal controls are a routine practice to identify invalid tests in LAMP assays (Nurul Najian, Engku Nur Syafirah, Ismail, Mohamed, & Yean, 2016). From those gill swabs analysed, only 1.6% of the tests were considered invalid. In the present study, the LAMP detection of the internal control was analysed in a separate tube than the LAMP detection of the pathogen. Due to the low ratio of the parasite DNA versus host DNA in the samples, the LAMP multiplexing for those genes was not successful (data not shown). Therefore, a separate assay (but run in parallel) for the target gene and the internal control was used for the analysis of the field samples.

Gill swabs are a resourceful non‐lethal sampling tool for AGD in Atlantic salmon. In the present study, a trend of negative correlation between the visual gill score and the *Tp* for the LAMP assay detection was observed. In previous studies, the gill score has shown a good correlation with histopathology scores and the molecular detection of *N. perurans* when using TaqMan™ qPCR assays (Downes et al., 2017). The developed LAMP assay performs very well (100% of positives) for swabs taken from fish showing gill scores ˃3. However, lower gill scores do require a bigger sample size to confirm the presence of *N. perurans* in non‐lethal gill swabs. Similarly, the molecular detection of *N. perurans* from gill swabs using real‐time PCR showed a decreased number of positives for lower gill scores (Downes et al., 2017). Therefore, for field sampling, the sample size should be calculated in advance considering the gill score. However, current practices to mitigate AGD on Atlantic salmon are based on early detection to inform site management (e.g. cage rotation) and treatment administration, typically consisting of freshwater or hydrogen peroxide bathing (Oldham et al., 2016). That early detection requires that an AGD‐POC test should be able, at least, to detect 100% of swabs taken from gill scores as low as 1. Given the analytical sensitivity of the current LAMP assay (LOD of one amoeba on the swab extract), it is disappointing that the current AGD‐POC test only detected the *N. perurans* 18S rRNA gene in ~50% of the swabs taken of low gill score. The assay can detect 100% of positives for low gill scores when using a reference DNA extraction method; thus, its poor performance in the field could be explained due an inefficient POC DNA extraction method. Therefore, future work should focus on improving POC DNA extraction methods to allow for the field deployment of this AGD‐POC test.

## 5 CONCLUSIONS

Overall, the simplicity, performance and low cost of the present AGD‐LAMP assay, with a gross estimated cost of £2.5 per sample (including the cost for DNA extraction and the isothermal amplification) versus £6.8 for a TaqMan™ qPCR assay, make this test a good candidate for the on‐site confirmation of *N. perurans* in non‐lethal gill swabs taken from Atlantic salmon clinically infected with AGD; however, further work is required to improve the parasite detection in low gill scores.

## CONFLICT OF INTEREST

The authors declare that the research was conducted in the absence of any commercial or financial relationships that could be construed as a potential conflict of interest.

## Supporting information

Supplement S1

Supplement S2

## Data Availability

The raw data presented in this manuscript are available upon request.
