# Supplementary material for: Non‐lethal loop‐mediated isothermal amplification assay as a point‐of‐care diagnostics tool for Neoparamoeba perurans, the causative agent of amoebic gill disease
Source: J Fish Dis. 2020 May 4;43(7):779–90. doi: 10.1111/jfd.13175 (PMC7383609; doi:10.1111/jfd.13175)
Supplement: Supplementary file 1 — Supplement S1 [file JFD-43-779-s001.docx]

**Supplement S1.** Standard curve derived from the correlation of the plasmid number copy and the *Ct* values in a Taqman™ qPCR assay. Slope-3.5; efficiency 91.4%; *r*^2^ 0.99.

**
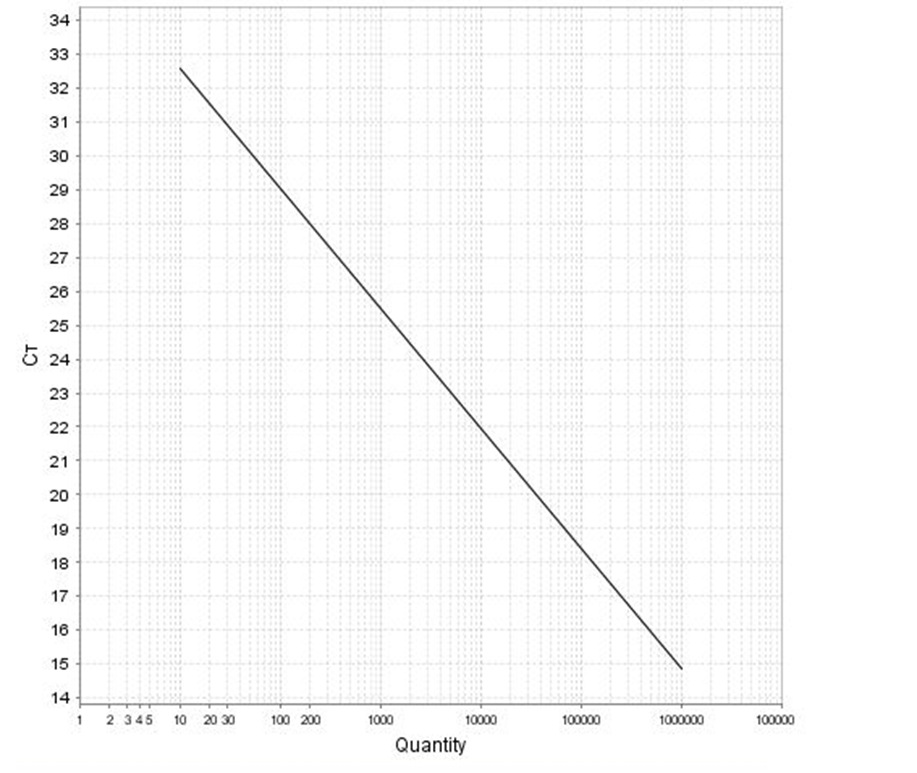
**
