# Supplementary material for: Non‐lethal loop‐mediated isothermal amplification assay as a point‐of‐care diagnostics tool for Neoparamoeba perurans, the causative agent of amoebic gill disease
Source: J Fish Dis. 2020 May 4;43(7):779–90. doi: 10.1111/jfd.13175 (PMC7383609; doi:10.1111/jfd.13175)
Supplement: Supplementary file 2 — Supplement S2 [file JFD-43-779-s002.docx]

**Supplement S2**

**Table S1** Point-of-care identification of *Neoparamoebae perurans* by LAMP assay. DNA was extracted using the QuickExtract method. The Atlantic salmon elongation factor 1a LAMP assay was used as internal control. LAMP detection expressed as time of positivity Tp (mm:ss). Anneal temperature (AT). The visual gill index was expressed as an average of the gill score for each hemibranch surface. Positives coloured in red. Invalid tesd coloured in blue.

| **Fish ID** | **Gill Score** | ***Tp N. Perurans*** | **AT (°C) *N. Perurans*** | ***Tp* Atlantic salmon** | **AT (°C) Atlantic salmon** | **Test result** |
| --- | --- | --- | --- | --- | --- | --- |
| **26** | 0.88 | 0 | 0.0 | 30:15 | 88.0 | Negative |
| **27** | 0.13 | 0 | 86.1 | 21:15 | 88.2 | Negative |
| **28** | 0.38 | 0 | 0.0 | 36:15 | 88.0 | Negative |
| **29** | 0.38 | 0 | 86.7 | 26:15 | 88.1 | Negative |
| **30** | 0.5 | 0 | 0.0 | 31:00 | 88.0 | Negative |
| **31** | 1.38 | 21:30 | 81.6 | 27:15 | 88.1 | Positive |
| **32** | 0.75 | 0 | 0.0 | 29:30 | 88.1 | Negative |
| **33** | 0.5 | 0 | 0.0 | 27:15 | 88.2 | Negative |
| **34** | 0.88 | 38:00 | 81.7 | 30:00 | 88.2 | Positive |
| **35** | 1 | 38:00 | 80.7 | 26:15 | 88.2 | Positive |
| **36** | 2.38 | 0 | 0.0 | 27:00 | 88.2 | Negative |
| **37** | 1.5 | 39:00 | 80.6 | 24:00 | 88.2 | Positive |
| **38** | 2.13 | 39:00 | 81.7 | 26:00 | 88.2 | Positive |
| **39** | 1.13 | 0 | 0.0 | 24:00 | 88.2 | Negative |
| **40** | 2.13 | 0 | 0.0 | 23:00 | 88.2 | Negative |
| **41** | 2.5 | 0 | 0.0 | 30:00 | 88.1 | Negative |
| **42** | 1.75 | 39:15 | 81.5 | 23:00 | 88.1 | Positive |
| **43** | 2 | 0 | 0.0 | 36:15 | 88.1 | Negative |
| **44** | 1.88 | 0 | 0.0 | 30:00 | 88.1 | Negative |
| **45** | 2 | 39:00 | 81.6 | 26:45 | 88.2 | Positive |
| **46** | 0.88 | 0 | 0.0 | 23:30 | 88.2 | Negative |
| **47** | 1 | 13:00 | 81.4 | 12:27 | 88.2 | Positive |
| **48** | 0.38 | 0 | 0.0 | 24:00 | 88.2 | Negative |
| **49** | 0.63 | 0 | 0.0 | 0 | 0.0 | Invalid test |
| **50** | 1.25 | 15:00 | 81.6 | 23:00 | 88.2 | Positive |
| **51** | 0.75 | 22:00 | 81.7 | 23:45 | 87.7 | Positive |
| **52** | 0.88 | 26:15 | 81.6 | 24:00 | 87.9 | Positive |
| **53** | 0.63 | 39:00 | 81.9 | 13:45 | 88.1 | Positive |
| **54** | 0.5 | 33:00 | 81.4 | 22:45 | 87.9 | Positive |
| **55** | 0.25 | 19:45 | 81.8 | 23:15 | 88.1 | Positive |
| **56** | 1.13 | 0 | 0.0 | 17:00 | 88.1 | Negative |
| **57** | 1 | 31:00 | 81.9 | 18:00 | 88.0 | Positive |
| **58** | 1.38 | 39:15 | 81.5 | 18:45 | 88.0 | Positive |
| **59** | 1.38 | 25:00 | 81.2 | 15:30 | 88.0 | Positive |
| **60** | 1.25 | 35:00 | 81.3 | 19:45 | 87.9 | Positive |
| **61** | 1.25 | 27:30 | 81.8 | 19:30 | 88.2 | Positive |
| **62** | 1 | 38 | 81.8 | 19:45 | 87.8 | Positive |
| **63** | 1.38 | 30:00 | 81.9 | 16:30 | 88.2 | Positive |
| **64** | 0.75 | 39 | 81.0 | 21:45 | 88.1 | Positive |
| **65** | 0.75 | 0 | 0.0 | 19:15 | 88.1 | Negative |
| **66** | 3.33 | 34:45 | 81.6 | 18:15 | 88.0 | Positive |
| **67** | 3.37 | 23:15 | 82.0 | 17:15 | 88.1 | Positive |
| **68** | 2.63 | 0 | 0.0 | 21:45 | 88.0 | Negative |
| **69** | 3.58 | 26:00 | 81.7 | 19:00 | 87.9 | Positive |
| **70** | 2.38 | 27:00 | 81.7 | 15:30 | 88.0 | Positive |
| **71** | 3.08 | 32:00 | 81.7 | 14:15 | 88.1 | Positive |
| **72** | 3.04 | 28:00 | 81.5 | 17:15 | 88.1 | Positive |
| **73** | 2.62 | 19:30 | 81.8 | 19:30 | 88.2 | Positive |
| **74** | 2.67 | 24:00 | 81.7 | 16:00 | 88.0 | Positive |
| **75** | 3.16 | 31:45 | 81.6 | 16:00 | 88.1 | Positive |
| **76** | 1.38 | 0 | 0.0 | 32:15 | 88.2 | Negative |
| **77** | 1.88 | 0 | 0.0 | 27:00 | 88.2 | Negative |
| **78** | 1.63 | 39:00 | 81.3 | 26:15 | 88.3 | Positive |
| **79** | 1.88 | 38:45 | 81.7 | 22:45 | 88.1 | Positive |
| **80** | 1.5 | 0 | 0.0 | 24:45 | 88.0 | Negative |
| **81** | 1.75 | 0 | 0.0 | 26:30 | 88.2 | Negative |
| **82** | 2.13 | 35:45 | 82.1 | 26:30 | 88.2 | Positive |
| **83** | 1.63 | 0 | 0.0 | 26:45 | 88.1 | Negative |
| **84** | 1.5 | 39:00 | 81.4 | 23:15 | 88.2 | Positive |
| **85** | 1.63 | 0 | 0.0 | 30:30 | 88.1 | Negative |

**Graphs S1.** Isothermal amplification of *Neoparamoebae perurans* from Isohelix swabs. For each fish, the amplification plot, amplification rate and anneal derivative is shown for both the *N. perurans* 18S rRNA gene and the Atlantic salmon elongation factor 1a (EF1a) LAMP assays. The Atlantic salmon EF1a LAMP assay was used as internal control. AGD-positive infected tissue was used as postive control in the *N. perurans* LAMP assay. Water was used as negative control.
